# Three-Dimensional *In vivo* Magnetic Resonance Imaging (MRI) of Mouse Facial Nerve Regeneration

**DOI:** 10.3389/fneur.2019.00310

**Published:** 2019-04-02

**Authors:** Renate Wanner, Alireza Abaei, Volker Rasche, Bernd Knöll

**Affiliations:** ^1^Institute of Physiological Chemistry, Ulm University, Ulm, Germany; ^2^Core Facility Small Animal MRI, Medical Faculty, Ulm University, Ulm, Germany; ^3^Department of Internal Medicine II, University Hospital Ulm, Ulm, Germany

**Keywords:** axon regeneration, facial nerve, MRI, peripheral nerve, mouse

## Abstract

MRI (magnetic resonance imaging) is an indispensable tool in the diagnosis of centrals nervous system (CNS) disorders such as spinal cord injury and multiple sclerosis (MS). In contrast, diagnosis of peripheral nerve injuries largely depends on clinical and electrophysiological parameters. Thus, currently MRI is not regularly used which in part is due to small nerve calibers and isointensity with surrounding tissue such as muscles. In this study we performed translational MRI research in mice to establish a novel MRI protocol visualizing intact and injured peripheral nerves in a non-invasive manner without contrast agents. With this protocol we were able to image even very small nerves and nerve branches such as the mouse facial nerve (diameter 100–300 μm) at highest spatial resolution. Analysis was performed in the same animal in a longitudinal study spanning 3 weeks after injury. Nerve injury caused hyperintense signal in T_2_-weighted images and an increase in nerve size of the proximal and distal nerve stumps were observed. Further hyperintense signal was observed in a bulb-like structure in the lesion site, which correlated histologically with the production of fibrotic tissue and immune cell infiltration. The longitudinal MR representation of the facial nerve lesions correlated well with physiological recovery of nerve function by quantifying whisker movement. In summary, we provide a novel protocol in rodents allowing for non-invasive, non-contrast agent enhanced, high-resolution MR imaging of small peripheral nerves longitudinally over several weeks. This protocol might further help to establish MRI as an important diagnostic and post-surgery follow-up tool to monitor peripheral nerve injuries in humans.

## Introduction

Peripheral neuropathies including peripheral nerve injuries of extremities and long-term complications of nerves inflicted by diabetes are frequent. They affect 2.5% of the general population and 8% of those above aged over 55 (1, 2). Non-invasive imaging technologies such as MRI (Magnetic Resonance Imaging) have improved diagnosis and management of various neurological diseases particularly those affecting the CNS such as stroke, MS and spinal cord injury (3). In contrast in the peripheral nervous system (PNS), MRI (also referred to as MR neurography) is less frequently used as diagnostic tool. This has multi-factorial reasons such as lack of specificity of signal changes, poor structural resolution and contrast of small nerves and fascicles as well as the burden of an additional invasive procedure by contrast agent (CA) injection (4–8). Nevertheless, MR neurography has been applied to several peripheral neuropathies including Carpal Tunnel Syndrome, plexus, and other traumatic peripheral nerve lesions and PNS tumors (9). Typically, un-injured peripheral nerves are surrounded by a thin layer of fat tissue and appear isointense or, in T_2_-weighted sequences, moderately hyperintense compared to muscle tissue. After injury, so-called Wallerian degeneration is induced in peripheral nerves including myelin debris clearance by Schwann cells and phagocytosing immune cells (7). In this case, injured nerves now frequently appear brighter in T_2_-weighted images. This initial signal hyperintensity in the early response phase after injury is often lost following successful nerve regeneration at much later time-points after injury (4–8). Such signal alterations between intact and damaged nerves may help to diagnose nerve damage *in vivo* and to follow nerve regeneration after surgery. MR neurography is further improved by contrast agents specifically accumulating in the nerve fibers such as gadofluorine M (5), however at the expense of a further invasive injection with potential complications for the patient.

In this study we aimed at establishing an MRI protocol in the mouse allowing to monitor regeneration of smallest nerves and nerve branches without application of contrast agents. So far, rodent models have been useful as a translational MRI model to successfully image several larger peripheral nerves such as the sciatic (10–12) or optic nerve (13–15). In this study we aimed toward investigating the facial nerve in adult mice, which represents one of the smallest peripheral nerves.

The facial nerve (FN) is divided into several ellipsoid branches ranging between 100 and 300 μm. Facial nerve paralysis is one of the most frequent peripheral nerve injuries in humans, thus novel high-resolution MRI protocols established in a rodent model system bear immediate translational potential to the clinics. The facial nerve branches in rodents connect facial neurons (FMN) located in the brainstem with facial muscles stirring e.g., eyelid closure and whisker movement (16). *In vivo*, mouse facial nerve regeneration was so far visualized with GFP labeling of axons (17) however not with MRI. In contrast to mice, MRI was successfully applied to image the larger rat (18) and rabbit (19) facial nerve.

We developed a high-resolution three-dimensional T_2_-weighted MRI sequence allowing to longitudinally visualize the same facial nerve branches of an individual mouse before and at several time-points within 3 weeks after injury. In accordance with previous findings, we observed nerve hyperintensity in the MR images after injury. We provide a cellular source for this hyperintensity by performing correlative immunehistological inspection, demonstrating accumulation of fibrotic material and several cell types including immune and Schwann cells in such hyperintense nerve areas. In summary, we provide a novel MRI protocol allowing for longitudinal high-resolution imaging of smallest nerve branches at identical positions in the same animal over several weeks.

## Materials and Methods

### Mice

Seven male adult wild-type C57BL/6J mice aged 13–14 weeks were enrolled in this study. All experiments in this study were reviewed and approved by and were in accordance with regulations by the local veterinary authorities (Regierungspräsidium Tübingen, Germany).

### Facial Nerve Trauma

For unilateral traumatic facial nerve trauma, adult mice were anesthetized with isoflurane inhalation. Prior to the injury, 50 mg/ml Temgesic was injected for analgesia. To expose the buccal and marginal branches of the FN, a skin incision was made in the area of the masseter muscle and transected with microscissors. The absence of whisker movement confirmed a successful facial nerve trauma.

### MRI

**Figure 1:**
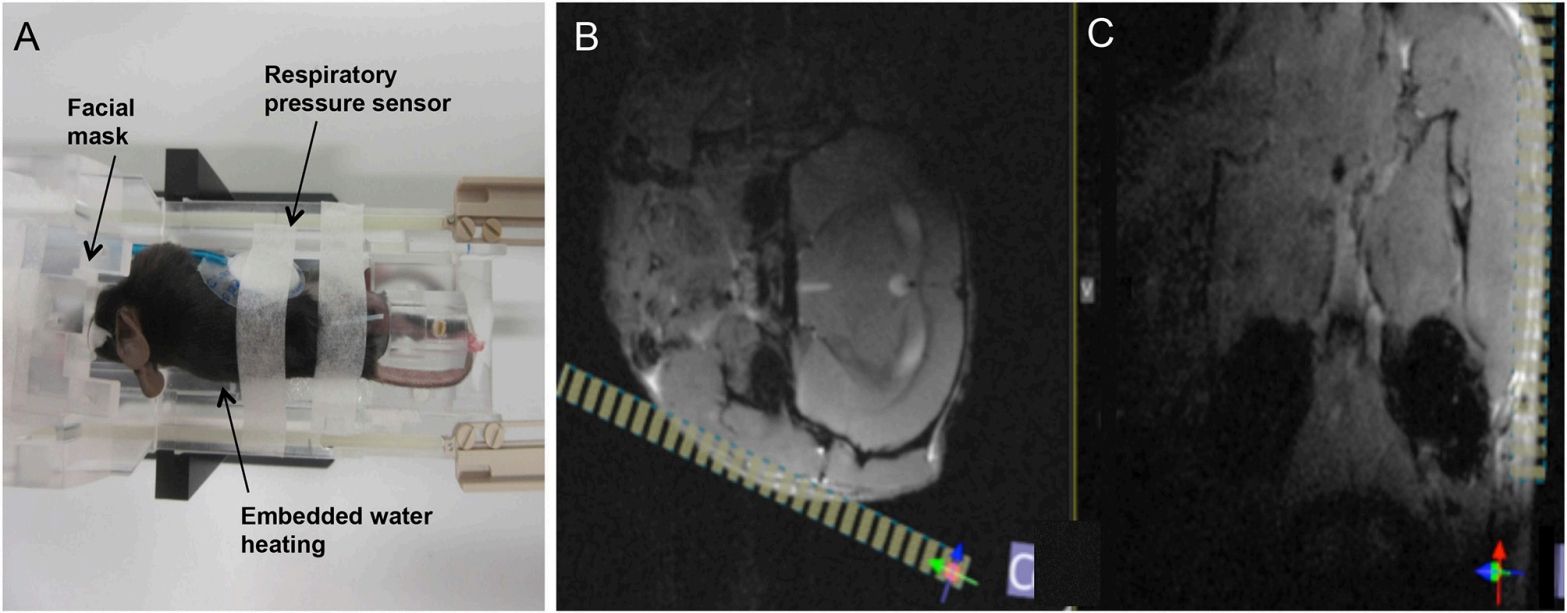
Overview on animal positioning and planning of the final scan volume. **(A)** The animal was placed in lateral position in the dedicated cryocoil animal support. Anesthesia gas was provided via a face mask, respiration frequency monitored by a pressure sensor, and the body temperature controlled by an embedded water heating system. **(B,C)** Final planning of the volume of interest (VOI, yellow stripes) was done on the axial **(B)** and coronal **(C)** FLASH images, based on anatomical landmarks (masseter, mandible) for ensuring coverage of both branches of the facial nerve.

MRI was performed at 1 day post injury (dpi), 5dpi, 9dpi, 13dpi, 16 dpi, and 21dpi, applying a standardized imaging protocol. Measurements were performed on an 11.7 T small animal MRI (BioSpec 117/16, Bruker Biospin). All data were acquired using a cryogenically-cooled 1H two-element surface transmit/receive coil (MRI CryoProbe™, Bruker BioSpec, Ettlingen, Germany). After initiation of the anesthesia with 5% isoflurane in air, the mice were placed in a lateral position in the cradle with the injured nerve facing the surface of the coil. The head was securely fixed by a three point fixation device (Figure 1A). The anesthesia gas was administered via a facial mask and during scanning, the isoflurane concentration was adjusted between 1.25 and 1.5% to maintain the respiratory frequency at about 90 cycles per minute.

After obtaining conventional low-resolution localizer data in axial, coronal and sagittal orientation, two multi slice gradient echo anatomical scans were performed in axial (FLASH axial) and coronal (FLASH coronal) orientations covering the entire brain of the mouse. Anatomical landmarks were identified as indicated in Figures 1B,C and the final high-resolution 3D scan volume planned orthogonal to the masseter muscle and almost parallel to the mandible to ensure coverage of the facial nerve bundle (Figures 1B,C). For final data acquisition, a three-dimensional high resolution fat-suppressed mildly T_2_-weighted RARE (T2W-3D-RARE) sequence, optimized for facial nerve delineation, was applied. Acquisition parameters for all sequences are summarized in (Table 1).

**Table 1:** Parameters of MR imaging acquisition.

	**T2W-3D-RARE**	**FLASH axial**	**FLASH Coronal**
Encoding	Three-dimensional	Multi-slice	Multi-slice
TR/TE [ms]	1750/30	360/5	193/2.4
Echo spacing [ms]	7.5	NA	NA
Readout bandwidth [kHz]	100	96	96
Resolution [μm3]	65 × 65 × 65	57 × 57 × 500	65 × 65 × 500
Matrix	245 × 245 × 15	350 × 350 × 33	260 × 260 × 19
Field of view [mm3]	16 × 16 × 0.975	20 × 20 × 16.5	17 × 17 × 9.5
NSA	1	1	1
Acquisition time [s]	785	126	50
Fat suppression	Spectral-selective BW = 1750 Hz	NA	NA

For data analysis, the three-dimensional MRI data slab was carefully aligned with the respective nerve branch under investigation by multi-planar reformatting (MPR, RadiAnt DICOM Viewer) to allow longitudinal assessment of the fiber bundle. For further inspection orthogonal cross-sectional views of the nerve were visualized at relevant locations along the nerve.

### Whisker Movement

Whisker movement analysis was performed as described previously (20). To accustom the mice for videotaping, the animals where handled prior the experiments. For videotaping, whiskers above and below the C whiskers were clipped in anesthetized mice by microscissors. Mice were hand restrained and videotaped by a high-speed camera (Basler acA1300–60 gc) for 51 s at 100 Hz from top view. 1 s video fragments were transferred to Templo Software (CONTEMPLAS GmbH, Germany) and analyzed by Vicon Motus 2D software (CONTEMPLAS GmbH, Germany). A spatial model, comprising a line between the right and the left eye as individual fix points and an associated mid-sagittal line in a 90° angle was used to determine the angular whisker positions. For the analysis, a whisker was defined in a line set up by a point at the whisker shaft and a point approximately 0.5 cm in proximity. The whisker position was analyzed toward the mid-sagittal plane for 1 s. The parameter range describes the difference between the maximum and the minimal displacement of the whisker within one time frame. We used 5 mice for 0, 13 and 21 dpi, 2 animals for 1 and 5 dpi and 3 animals for 3, 6 and 16 dpi.

### Fluorescent Tracer Injection

Fluorescent tracers were applied as described previously (20, 21). The quantification of nerve regeneration was visualized by the retrograde tracers 1,1′-dioctadecyl-3,3,3′,3′-tetramethylindocarbocyanine perchlorate (DiI; Molecular Probes), fluorogold (FG; fluorochrome) and cholera toxin subunit B Alexa fluor 488 conjugate (Ctx488; Invitrogen). Mice were anesthetized and 50 mg/ml Temgesic was injected. In the eye, whisker pad and lower lip, 2 × 1 μl of DiI (2 μg/μl DMSO), 4 × 1 μl FG (4% in H_2_O) and 2 × 1 μl Ctx488 (1 μg/μl in PBS) were injected with a Hamilton syringe. The injection was performed directly after the last MRI measurement and the mice were sacrificed 1 day later. The brains were fixed in 4% paraformaldehyde (PFA), embedded in agarose and cut with a vibratome into 80 μm thick sections. For each animal, both facial nuclei were analyzed with four sections. FG-, DiI and Ctx488-positive neurons were quantified manually in ImageJ.

### Immunostaining

Masseter muscles were dissected and stored in 30% sucrose/PBS ON at 4°C. Following, the tissue was embedded in Tissue-Tek O.C.T. (optimal cutting temperature) compound and frozen by nitrogen-cooled isopentane. Embedded sections were cut into 10 μm thick sections with a cryotome. Prior immunohistochemistry, the sections were post-fixed in 4% PFA for 10 min. For primary antibodies, anti-βIII-tubulin (mouse, 1:2000, Covance, MMS-435P), anti-S100β (rabbit, 1:1000, Abcam, ab52642), Vimentin (rabbit, 1:130, Abcam, ab 7783) and CD45 (rat, 1:1000, BD Pharmingen, 550539) were used. For detection of the primary antibodies, Alexa Fluor 488- or 546 conjugated secondary antibodies were used.

### Quantification of MRI Data

Transverse nerve size measurements were performed manually in RadiAnt DICOM Viewer 4.2.1 evaluation version. For the proximal and distal nerve stumps, the upper marginal branches were measured in 0.25 mm range to the injury sides by 3 measurements. The three values were averaged. The nerve thickness for the gap was gained by measuring the entire marginal nerve before injury and from the 9 dpi on by 3 measurements within a 0.25 mm range for each time point. The values were averaged. The mean intensity of the nerves was measured in ImageJ manually. The mean intensity was determined by the ratio of the mean intensity of the upper marginal nerve toward the value of the adjacent muscle. For the gap, nerve-mean intensity was compared to muscular mean intensity, averaged from proximal and distal muscular intensity.

### Statistical Analysis

Statistical analysis was performed with the GraphPad Prism software. The type of statistical test is also indicated in the figure legend. Statistical significance is provided as ^*^, ^**^, ^***^ indicating p ≤ 0.05, 0.01 and 0.001, respectively and “ns” signifies “not significant.” All data is depicted as mean ± SD if not indicated otherwise.

## Results

### Establishing a MRI Sequence to Image the Intact Facial Nerve of Adult Mice

**Figure 2:**
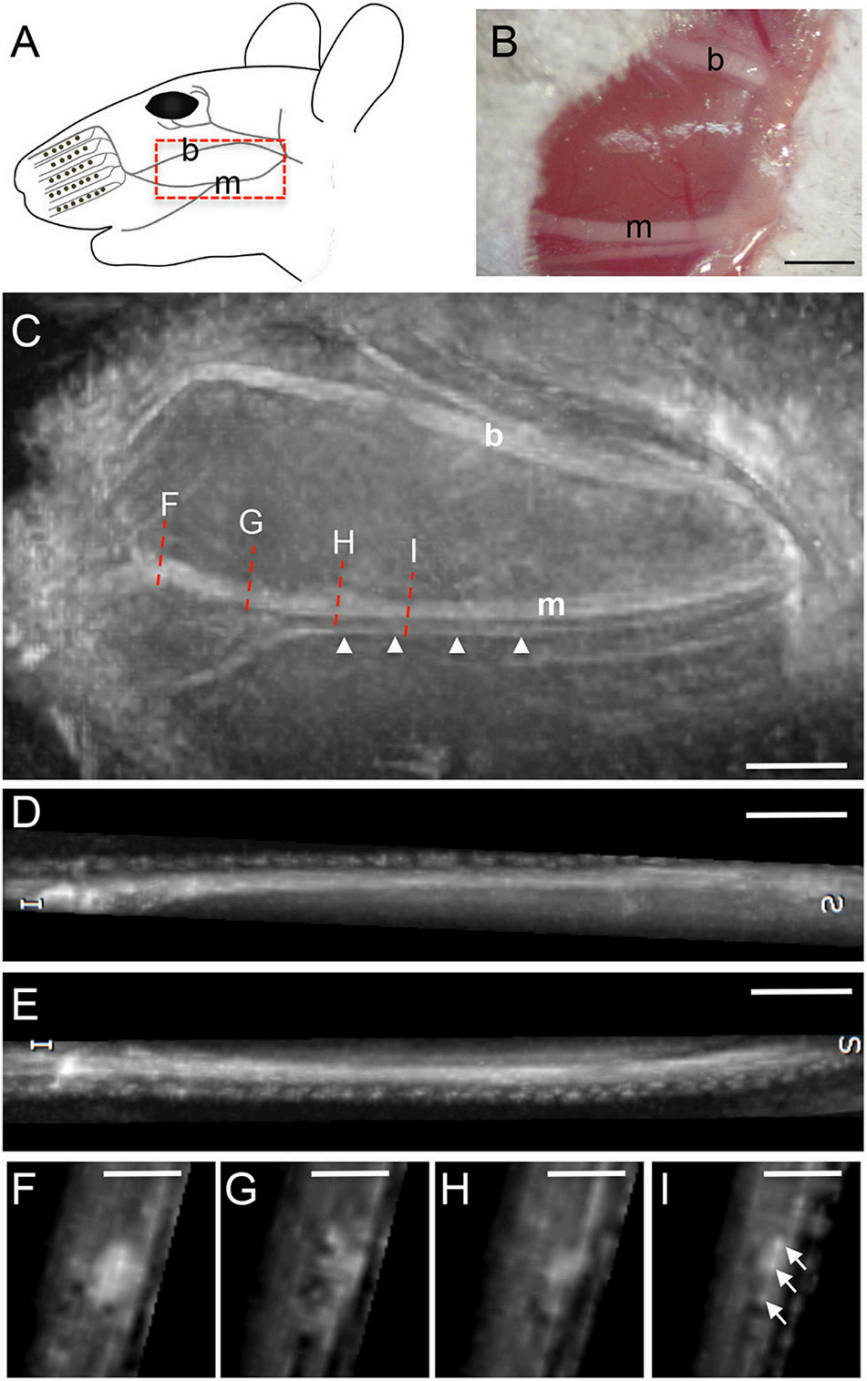
MR imaging of the intact mouse facial nerve. **(A)** Scheme of facial nerve branches including the buccal (b) and marginal (m) branch in the adult mouse. **(B)** Light microscopical picture of the buccal and marginal facial nerve branches before MR imaging. Both nerves appear as white structures localized on top of the masseter muscle. **(C)** Fat-suppressed, T_2_-weighted MR image depicting the buccal and marginal branch in rostro-caudal slice orientation. The orientation of the picture is identical to the scheme in **(A)**. Both nerve branches are hyperintense compared to the surrounding muscle tissue. The marginal branch is split in a thicker and a thinner branch (arrowheads) with approximately only 100 μm or less in thickness. The red dotted lines indicate the four positions of cross-section through the nerve provided in **(F–I)**. **(D,E)** Representation of the buccal **(D)** and marginal **(E)** branch in longitudinal orientation, clearly showing the thickness variation of the respective nerves along its course. **(F–I)** Four cross-sections at different rostro-caudal positions of the marginal branch in **(C)**. Several smaller fascicles of the marginal branch are visible in **(I)**. Scale-bar = 1 mm **(B–E)**, 0.5 mm **(F–I)**.

The facial nerve in mice is divided into several branches including the buccal (b) and marginal (m) branch [highlighted in red dotted box; Figure 2A; (16)] innervating the whisker pad. The marginal branch is divided in a bigger major and a smaller minor branch (Figure 2B). In addition to MR imaging, both facial nerve branches localized on top of the masseter muscle were photographed (Figure 2B). Subsequently, the animal was positioned in the scanner for imaging of the facial nerve branches (Figures 2C–I). Both, the buccal branch and the two rami of the marginal branches were visualized at high resolution. Notably, the minor branch of the marginal facial nerve has a width of >100 μm and was resolved at high resolution (arrowheads, Figure 2C; see materials and methods for resolution). Both branches could be followed over a distance of at least 7–8 mm thereby allowing to monitor nerve structure over a considerably long distance (Figures 2C,D,E). Sagittal reformats clearly show the capability of the isotropic three-dimensional MRI to even assess the thickness of the buccal (D) and marginal (E) branch. With the chosen sequence timing, all nerve branches could be clearly delineated from the surrounding tissue by its hyperintense signal (Figures 2C–E). The signal intensity within one nerve branch also varied for instance along the rostro-caudal axis (see Figures 2C–E). This was also obvious from various nerve cross-sections (Figures 2F–I) taken at several positions along the rostro-caudal axis (red dotted lines in Figure 2C). On nerve cross-sections resolution was sufficient to observe dot-like structures (arrows Figure 2I) that might represent fascicles within one nerve branch. Please note some residual fat signal at the tissue air interfaces caused by the respective strong susceptibility transition.

### Longitudinal MRI Monitoring of Mouse Facial Nerve Regeneration Over 3 Weeks

**Figure 3:**
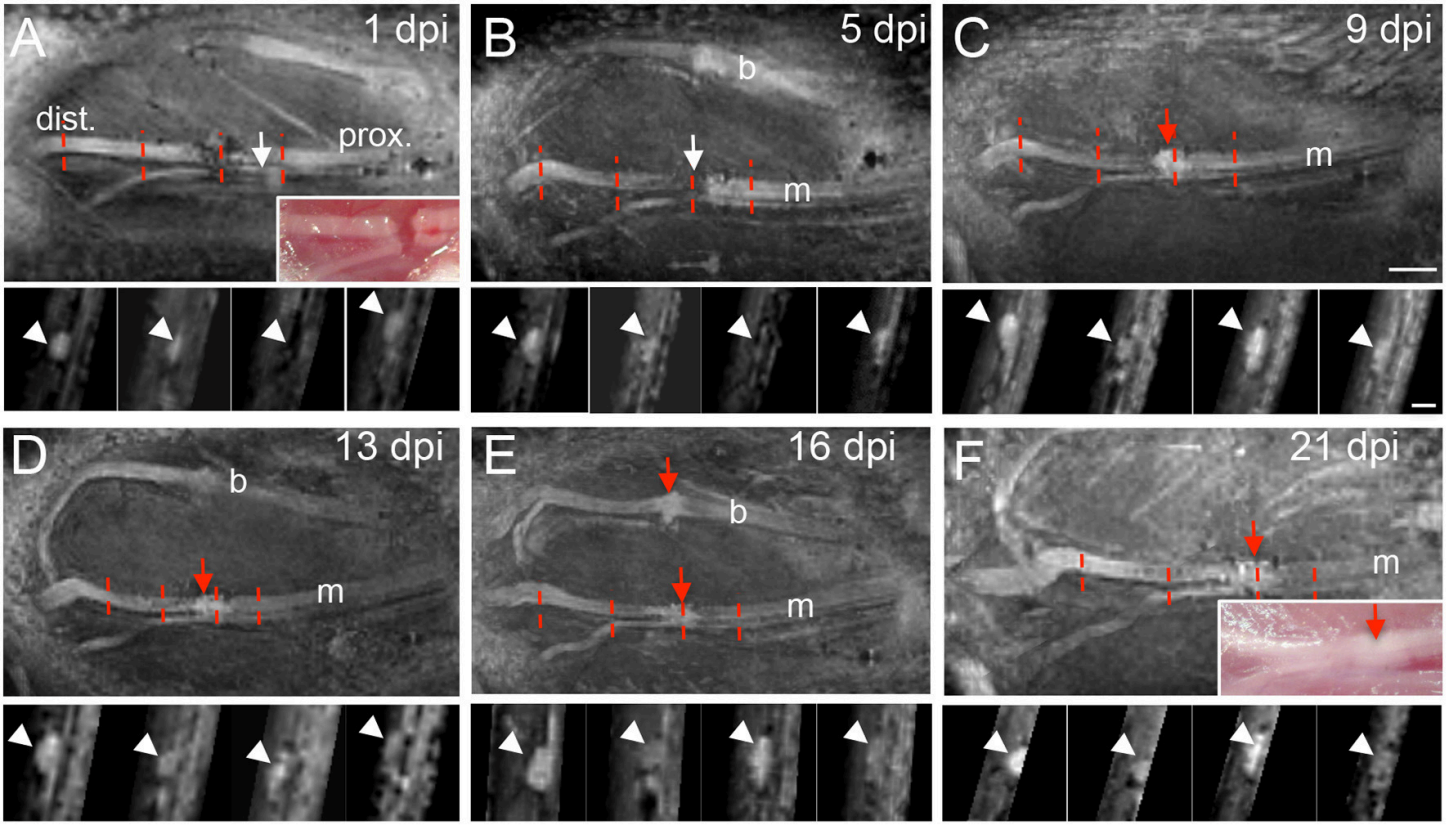
MR imaging of the injured mouse facial nerve. **(A–F)** T_2_-weighted images showing the facial nerve at 1 **(A)**, 5 **(B)**, 9 **(C)**, 13 **(D)**, 16 **(E)**, and 21 **(F)** days post injury (dpi). In **(A)** and **(F)** light microscopical pictures corresponding to the MRI pictures are provided as inserts. The position of the distal (“dist.”) and proximal (“prox.”) nerve stump is depicted in **(A)** and is identical for all pictures (see also Figure 1A for orientation). White arrows in **(A,B)** point at the hypointensity at the position of the lesion gap between both nerve parts. At 9 days post injury, a hyperintensity is present at the lesion site [red arrow in **(C)**] that persists also at the later injury time-points [red arrows in **(D–F)]**. In some pictures **(A,B,D,E)** the buccal and marginal branches are visible. The red dotted lines in **(A–F)** represent four positions of nerve cross-sections provided underneath each sagittal MRI picture. Arrowheads point at the position of the facial nerve on the cross-sections in **(A–F)**. *N* = 5 animals. Scale-bar = 1mm (**A–F**, main pictures), 0.5 mm (inserts in **A–F**).

In the next series of experiments the buccal and marginal branches of the facial nerve were completely cut with scissors (Figure 3). We only injured the facial nerve on one side of each animal, thus, the contralateral facial nerve on the other side of the face was spared and served as control within the same animal. Injury separated the nerve into a distal (“dist.”; Figure 3A) nerve stump without connection to the FMN cell bodies and a proximal (“prox.”; Figure 3A) nerve part still connected to the brainstem cell bodies. This injury site was photographed immediately after injury (insert, Figure 3A). Subsequently, the skin overlaying the lesion site and masseter muscle was sutured and MR recording started. We focused on axonal regeneration of the marginal branch by imaging the same animal at one, five, nine, 13, 16, and 21 days post injury (dpi; Figure 3). At each time-point, one view of the nerve along the rostro-caudal axis is provided together with four cross-sections at several positions along the sagittal axis (indicated by red dotted lines in Figure 3).

**Figure 4:**
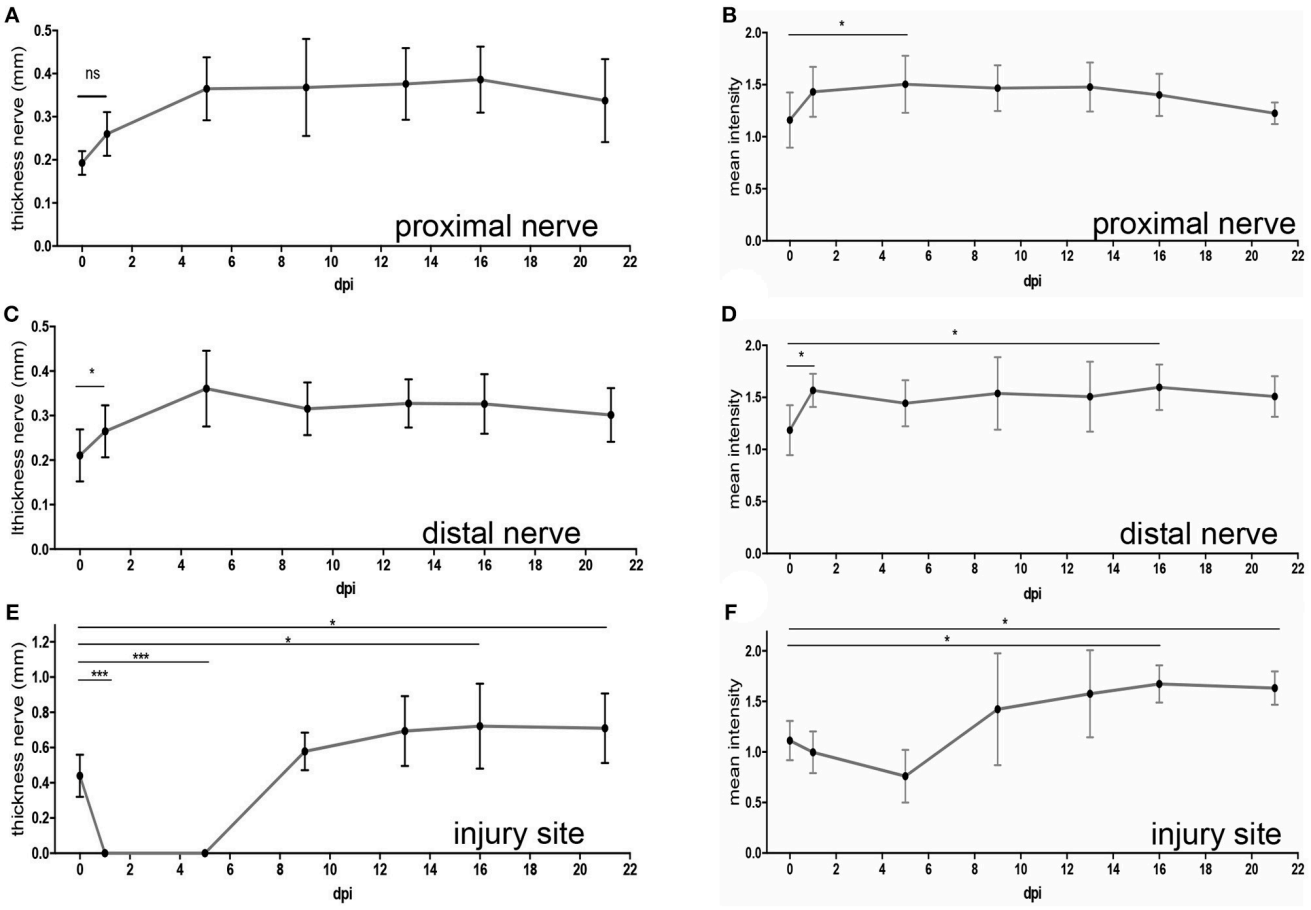
Quantification of MR imaging after mouse facial nerve injury. Quantification of the nerve thickness **(A,C,E)** or mean MRI signal intensity **(B,D,F)** of the proximal **(A,B)** and distal **(C,D)** nerve stump as well as the injury site **(E,F)**. Intensities were measured in the lesion center and, for proximal and distal nerves, 250 μm away from injury core. The time-point “0” depicts values before injury. In addition, several time-points post injury are shown. Already starting at 1dpi, nerve thickness and MRI signal intensity was increased for both the proximal and distal nerve stumps **(A–D)**. At the injury site, the nerve thickness **(E)** and MRI signal **(F)** decreased immediately after injury. However, increased nerve thickness and signal hyperintensity was observed from 9 dpi onwards. Statistical significance was tested with a one-way ANOVA except for **(C)** where a Kruskal-Wallis test was used.

At one (Figure 3A) and five (Figure 3B) dpi we noted a clear hypointensity localized at the gap separating both nerve stumps (white arrows in Figures 3A,B). This lack of signal at the injury site was also reflected when quantifying a reduced mean nerve thickness (Figure 4E) and reduced T_2_-weighted MRI signal (Figure 4F) at this position (N ≥ 5 animals). In opposite to the injury site, from one dpi onwards a hyperintensity was quantified at the proximal (Figure 4B) and distal (Figure 4D) nerve stumps compared to the pre-injury condition. Similar to this hyperintensity, the nerve thickness increased at the proximal (Figure 4A) and distal (Figure 4C) nerve branch from ~200 μm up to 400 μm. Starting at 9 dpi we noted the development of a strong hyperintensity now present at the injury site (red arrow, Figure 3C and quantified in Figure 4F). The area of this signal also exceeded the nerve thickness present before injury application by about 2-fold (see quantification in Figure 4E) and resembles a “bulb-like” enlargement. This hyperintense signal was also present at 13 dpi (Figure 3D) and 16 dpi where the buccal and marginal facial nerve branch was detected (red arrows Figure 3E). Indeed, the signal increase in this bulb-like structure in the injury site persisted up until the latest time-point of imaging, i.e., 3 weeks after injury (Figure 3F). This was also corroborated by quantification of nerve thickness (Figure 4E) and intensity (Figure 4F) of several animals (*N* ≥ 5 animals).

Thus, in summary we observed hyperintensity of the proximal and distal nerve parts after injury. In addition, an expected signal loss at the injury site early after lesion was at later post-injury time-points replaced by appearance of a strong hyperintense signal.

### Correlative Histological and Functional Inspection of MR Imaged Animals

**Figure 5:**
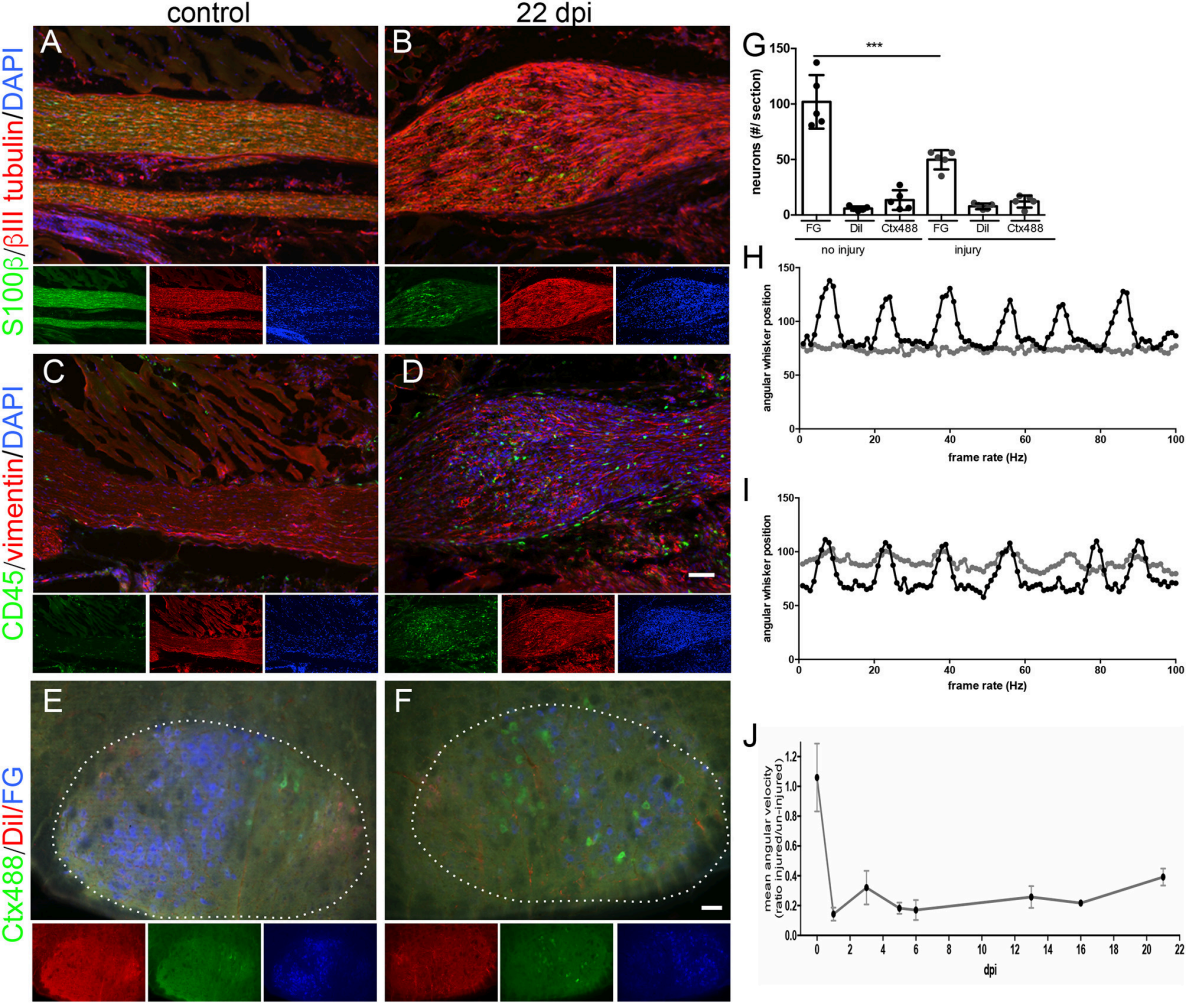
Correlative histological and functional analysis of MR imaged mice. **(A–D)** Histological sections of the intact [(**A,C)**; *N* = 3 animals] or injured [22 dpi; **(B,D)**; *N* = 5 animals] mouse facial nerve stained for expression of S100β to label Schwann cells (green in **A,B**) and of βIII tubulin to label axons (red in **A,B**), CD45-positive immune cells (green in **C,D**) or of vimentin to mark fibrotic material (red in **C,D**). DAPI staining (blue) labels all nuclei in **(A–D)**. Smaller pictures represent single channels of the main merged picture. At 22 dpi the nerve shows a bulb-like thickening **(B,D)** not present in the intact nerve **(A,C)**. After injury, Schwann cells **(B)**, CD45-positive immune cells **(D)** and more fibrotic material **(D)** is present in this bulb-like structure. **(E–G)** The fluorescent tracers Ctx488, DiI and FG are localized upon retrograde axonal transport in MN nucleus (white dotted line) in the brainstem (*N* = 5 animals). Without injury **(E)**, MNs of the same connectivity are topographically organized as revealed by grouping of MNs with the same color. At 22 dpi, number of fluorescently-labeled MNs has decreased indicative of incomplete regeneration **(F)**. Also, MNs are spread throughout the MN nucleus (borders indicated by white dotted line in **F**). **(G)** Quantification of absolute MN numbers positive for each fluorescent tracer before injury and at 22 dpi. Each circle depicts one animal analyzed. One-way ANOVA was used. **(H–J)** Analysis of whisker movement at one **(H)** and 22 **(I)** dpi. At one dpi, the whiskers connected by an injured nerve (gray line in **H**) do not oscillate whereas the whiskers on the intact contralateral side of the animal show a rhythmic pattern (black line in H). At 22 dpi, whisker movement triggered by the injured nerve (gray line in **I**) has partially recovered **(J)**. A ratio of whisker movement is provided between whiskers of both sides of the animal. Without injury, both whiskers move identically thus resulting in a ratio almost approaching 1. At one dpi this ratio decrease to approximately 0.1 and recovers to 0.4 at 22 dpi. Scale bar = 100 μm **(A–D)**, 100 μm **(E-F)**.

In MR imaging we observed at several time-points post lesion development of a hyperintensive signal at the lesion site (Figures 3, 4). Next, we wanted to correlate this signal with cellular processes to uncover a potential underlying cellular source for this signal. For this, animals were sacrificed after the last MRI session at 22 dpi and tissue was prepared for immune-histological examination (Figure 5). We analyzed the morphology of the injury site in comparison to an un-injured nerve (control) by staining βIII tubulin positive axons, S100β-positive Schwann cells (Figures 5A,B), infiltration of the lesion site by peripheral CD45-positive inflammatory cells and fibrosis by vimentin production (Figures 5C,D).

Compared to the thickness of the un-injured nerve (Figure 5A), a clear bulb-like extension of the nerve at the injury site was noticed similar to the MR image (compare e.g., Figure 3C with Figure 5B). Schwann cells were present in this bulb-like structure (green cells in Figure 5B). Similar to Schwann cells, the nerve injury site was infiltrated by CD45-positive immune cells (green cells in Figure 5D) that were absent in the control nerve (Figure 5C). Furthermore, vimentin signals indicative of tissue fibrosis accumulated more strongly in the injured nerve (red signals, Figure 5D) compared to the control nerve (Figure 5C). In general, we observed elevated cell numbers in the injured compared to control nerve as revealed by the presence of more DAPI-positive nuclei (Figures 5A–D).

In order to quantify the extent of functional axon regeneration we assessed two parameters, (i) retrograde axonal transport of fluorescent tracers at 22 dpi (Figures 5E–G) and (ii) recovery of whisker movement at 21 dpi (Figures 5H–J).

First of all, we injected fluorescent tracers (FG, Ctx488 and DiI) in three innervated muscle groups (whisker, lip and eyelid) at 21 dpi and allowed 1 day for tracer transport (resulting in 22 dpi). Upon successful re-growth of axon terminals to the respective muscles, fluorescent tracers are taken up and are retrogradely transported to the FMN cell bodies in the brainstem. In the brainstem, numbers of tracer-positive FMNs were quantified and compared to the respective contralateral un-injured FMNs of the same animal at 22 dpi (Figures 5E–G). In an un-injured animal, FMNs are typically topographically organized: FG-positive FMNs (blue in Figure 5E) representing the majority of axons innervating the whisker pad are restricted to the left half of the MN nucleus (white dotted line in Figure 5E). In contrast, eyelid and lip were represented by fewer FMNs and these FMNs were localized to the right half of the FMN nucleus (red and green in Figure 5E). At 22 dpi, the overall number of tracer-positive FMNs was reduced and they were randomly spread in the FMN nucleus (Figure 5F). For instance, quantification revealed that about only half the number of FG-positive FMNs were present at 22 dpi compared to the un-injured control (Figure 5G).

Second, we analyzed functional recovery of whisker movement along several time-points after injury (one, three, five, six, 13, 16, 21 dpi; Figures 5H–J). In the same animal, whisker movement was quantified for the un-injured control side and the injured facial nerve. One day after injury, the un-injured nerve of this animal showed the typical rhythmic pattern of whisker movement with several oscillations with approximately the same amplitude (black curve in Figure 5H). In contrast, whiskers on the injured side did not show any movement (gray curve in Figure 5H). Quantification revealed a mean angular velocity of approximately 0.1 at 1 dpi, thus achieving only 10% of the pre-injury status of whisker movement set to 1 (Figure 5J). At 21 dpi, we noted a reproducible recovery of whisker movement (gray curve in Figure 5I). Now, oscillations started again although not with the same amplitude as present for the intact whisker pad (Figure 5I). Quantification revealed that animals reached at 21 dpi approximately 40% (value: 0.39) of the pre-injury status (set to 1; Figure 5J).

In summary, the latest time-point of MR imaging (21 dpi) reflects conditions where about 50% of all axons have regenerated and re-innervated the whisker pad. Furthermore, whisker function was reconstituted to 40% of the maximal performance.

## Discussion

In this study we achieved imaging of the facial nerve and associated branches in the mouse. The facial nerve is one of the smallest peripheral nerves that so far has not been successfully imaged with MRI in the mouse. In the study, we focused on providing isotropic sub-1003 μm3 spatial resolution for enabling anatomic assessment of nerve branches down to a thickness of 100 μm or even below. To further improve delineation of the nerve structures, a mildly T_2_-weighted contrast was chosen providing hyperintense signal of the nerve structures (Figures 2, 3). Of note, this resolution and contrast was obtained without further injection of contrast agents, which is a clear advantage over CA-enhanced techniques for clinical translation. Still, contrast agents such as manganese or gadofluorine M were successfully applied to enhance resolution and contrast in pre-clinical PNS injury models (22). Thus, in a future study, it will be interesting to see whether isotropic high-resolution MRI protocols as established in this study results in further improved delineation of the nerves when injecting contrast agents or combined with further spin preparation techniques known to be sensitive to demyelination such as magnetization transfer contrast (MTC).

In agreement with previous reports (4–8) T_2_-weighted MRI signals of an un-injured peripheral nerve were isointense to mildly hyperintense relative to the surrounding muscle tissue (Figure 2). After facial nerve injury we also observed nerve thickening and elevated T_2_ signals starting at 1 dpi (Figures 3, 4). In previous reports such hyperintensity was reported for both, the proximal and distal nerve stump, however T_2_ signal elevation was more pronounced distally (22–24). In our study, a comparable signal increase in both nerve stumps was quantified (Figure 4) which might be due to the fact that measurements were performed in close vicinity to the injury center and not further away (see materials and methods). In addition, due to the higher spatial resolution we might have less problems with partial volume effects.

At the injury site we observed a bi-phasic response of the T_2_-weighted signal. Immediately after injury, there was an expected sharp drop in signal intensity due to nerve gap induced physical nerve separation (Figures 3A, B, 4F). However, from 9 dpi onwards we observed a strong hyperintensity restricted to a “bulb-like” structure in the injury site persisting the entire 3 weeks post-injury period (Figure 3). A similar structure was described after rat sciatic nerve injury (22). Thus, induction of a hyperintense structure in the lesion site might be a conserved mechanism in injured peripheral nerves. In previous studies, induction of hyperintensity is frequently followed by return of T_2_-weighted signals toward lower or baseline values at late time-points after injury (e.g., 2 month or later). This signal decrease is frequently accompanied by functional e.g., locomotor improvement (22, 24, 25). In our study, we did not observe such a drop in hyperintensity neither in the distal or proximal nerve stumps nor in the injury site (Figures 3, 4). Similarly, functional nerve regeneration was far from complete and ranged between 40 and 50% of functionality of the pre-injury status (Figure 5). However, compared to other studies in pre-clinical animal models we only analyzed 3 weeks after injury. Thus, it will be important to further extend the post-injury timespan to two-three month to correlate MRI signals with histological and functional nerve regeneration read-outs. Overall, others and we described in pre-clinical PNS injury models robust T_2_-weighted signal intensity changes and morphological hallmarks in the injured nerve but also with by the appearance, persistence and a potential dis-appearance of a bulb-like structure in the injury site. This suggests that reliable measurements of T_2_-weighted signals and morphological nerve changes resolved at high resolution in patients might be useful for monitoring regenerative success of disrupted nerves in the clinics.

The molecular nature and specificity of these MRI signal changes are debated since nerve de- and regeneration processes occur to some degree simultaneously and both might contribute to signal alterations (3). From a cellular perspective, myelin turnover, changes in the blood-nerve barrier, and axoplasmic flow caused by axonal degeneration, increased water content by an enlarged endoneurial space or entry of inflammatory cells could account for MRI signal changes (24, 25). In our study we observed elevated presence of DAPI-positive cells in the hyperintense bulb-like structure formed at the injury site (Figure 5). We identified the nature of these cells as Schwann cells, CD45-positive immune cells and fibrotic cells present at 22 dpi (Figure 5). Thus, our data suggest that elevated numbers of these cell types accumulating in the injured nerve stumps and injury site might contribute to the T_2_-weighted MRI hyperintensity observed in peripheral nerves after injury.

## Author Contributions

RW and AA performed all experiments and analyzed data. AA established the MRI method. VR and BK supervised the study and analyzed data. BK wrote the manuscript.

### Conflict of Interest Statement

The authors declare that the research was conducted in the absence of any commercial or financial relationships that could be construed as a potential conflict of interest.

## References

[1] England JD Asbury AK. Peripheral neuropathy. Lancet. (2004) 363:2151–61. 10.1016/S0140-6736(04)16508-2 15220040

[2] Sheikh KA. Non-invasive imaging of nerve regeneration. Exp Neurol. (2010) 223:72–6. 10.1016/j.expneurol.2009.07.008 19616546 PMC2849841

[3] Stroman PW Bosma RL Kornelsen J Lawrence-Dewar J Wheeler-Kingshott C Cadotte D. Advanced MR imaging techniques and characterization of residual anatomy. Clin Neurol Neurosurg. (2012) 114:460–70. 10.1016/j.clineuro.2012.01.003 22326716

[4] Bendszus M Stoll G. Technology insight: visualizing peripheral nerve injury using MRI. Nat Clin Pract Neurol. (2005) 1:45–53. 10.1038/ncpneuro0017 16932491

[5] Stoll G Bendszus M Perez J Pham M. Magnetic resonance imaging of the peripheral nervous system. J Neurol. (2009) 256:1043–51. 10.1007/s00415-009-5064-z 19252774

[6] Thawait SK Wang K Subhawong TK Williams EH Hashemi SS Machado AJ. Peripheral nerve surgery: the role of high-resolution MR neurography. AJNR Am J Neuroradiol. (2012) 33:203–10. 10.3174/ajnr.A2465 21527571 PMC7964800

[7] Ohana M Moser T Moussaoui A Kremer S Carlier RY Liverneaux P. Current and future imaging of the peripheral nervous system. Diagn Interv Imaging. (2014) 95:17–26. 10.1016/j.diii.2013.05.008 24144933

[8] Rangavajla G Mokarram N Masoodzadehgan N Pai SB Bellamkonda RV. Noninvasive imaging of peripheral nerves. Cells Tissues Organs. (2014) 200:69–77. 10.1159/000369451 25766202 PMC4494672

[9] Kollmer J Bendszus M Pham M. MR neurography: diagnostic imaging in the PNS. Clin Neuroradiol. (2015) 25 (Suppl. 2):283–9. 10.1007/s00062-015-0412-0 26070607

[10] Bendszus M Koltzenburg M Wessig C Solymosi L. Sequential MR imaging of denervated muscle: experimental study. AJNR Am J Neuroradiol. (2002) 23:1427–31. 12223392 PMC7976256

[11] Bendszus M Wessig C Schutz A Horn T Kleinschnitz C Sommer C. Assessment of nerve degeneration by gadofluorine M-enhanced magnetic resonance imaging. Ann Neurol. (2005) 57:388–95. 10.1002/ana.20404 15732113

[12] Lehmann HC Zhang J Mori S Sheikh KA. Diffusion tensor imaging to assess axonal regeneration in peripheral nerves. Exp Neurol. (2010) 223:238–44. 10.1016/j.expneurol.2009.10.012 19879260 PMC3038603

[13] Sandvig A Sandvig I Berry M Olsen O Pedersen TB Brekken C. Axonal tracing of the normal and regenerating visual pathway of mouse, rat, frog, and fish using manganese-enhanced MRI (MEMRI). J Magn Reson Imaging. (2011) 34:670–5. 10.1002/jmri.22631 21769959

[14] Haenold R Herrmann KH Schmidt S Reichenbach JR Schmidt KF Lowel S. Magnetic resonance imaging of the mouse visual pathway for *in vivo* studies of degeneration and regeneration in the CNS. Neuroimage. (2012) 59:363–76. 10.1016/j.neuroimage.2011.07.069 21835252

[15] Wang WL Xu H Li Y Ma ZZ Sun XD Hu YT. Dose response and time course of manganese-enhanced magnetic resonanceimaging for visual pathway tracing *in vivo*. Neural Regen Res. (2016) 11:1185–90. 10.4103/1673-5374.187065 27630707 PMC4994466

[16] Moran LB Graeber MB. The facial nerve axotomy model. Brain Res Brain Res Rev. (2004) 44:154–78. 10.1016/j.brainresrev.2003.11.004 15003391

[17] Placheta E Wood MD Lafontaine C Frey M Gordon T Borschel GH. Macroscopic *in vivo* imaging of facial nerve regeneration in Thy1-GFP rats. JAMA Facial Plast Surg. (2015) 17:8–15. 10.1001/jamafacial.2014.617 25317544

[18] Sutherland D Buist R Dort JC. Magnetic resonance imaging of the extratemporal facial nerve of the rat. J Otolaryngol. (1997) 26:112–5. 9106086

[19] Orloff LA Duckert LG. Magnetic resonance imaging of intratemporal facial nerve lesions in the animal model. Laryngoscope. (1995) 105(5 Pt 1):465–71. 10.1288/00005537-199505000-00002 7760658

[20] Wanner R Gey M Abaei A Warnecke D de Roy L Durselen L. Functional and molecular characterization of a novel traumatic peripheral nerve-muscle injury model. Neuromolecular Med. (2017) 19:357–74. 10.1007/s12017-017-8450-1 28689354

[21] Gey M Wanner R Schilling C Pedro MT Sinske D Knoll B. Atf3 mutant mice show reduced axon regeneration and impaired regeneration-associated gene induction after peripheral nerve injury. Open Biol. (2016) 6:160091. 10.1098/rsob.160091 27581653 PMC5008009

[22] Li HJ Zhang X Zhang F Wen XH Lu LJ Shen J. Enhanced repair effect of toll-like receptor 4 activation on neurotmesis: assessment using MR neurography. AJNR Am J Neuroradiol. (2014) 35:1608–14. 10.3174/ajnr.A3977 24874529 PMC7964434

[23] Titelbaum DS Frazier JL Grossman RI Joseph PM Yu LT Kassab EA. Wallerian degeneration and inflammation in rat peripheral nerve detected by *in vivo* MR imaging. AJNR Am J Neuroradiol. (1989) 10:741–6. 2549771 PMC8332617

[24] Behr B Schnabel R Mirastschijski U Ibrahim B Angenstein F Schneider W. Magnetic resonance imaging monitoring of peripheral nerve regeneration following neurotmesis at 4.7 Tesla. Plast Reconstr Surg. (2009) 123:1778–88. 10.1097/PRS.0b013e3181a3f343 19483579

[25] Cudlip SA Howe FA Griffiths JR Bell BA. Magnetic resonance neurography of peripheral nerve following experimental crush injury, and correlation with functional deficit. J Neurosurg. (2002) 96:755–9. 10.3171/jns.2002.96.4.0755 11990818

